# Strain-specific morphology and CNS propagation of template-directed SOD1 aggregation following peripheral nerve inoculation

**DOI:** 10.1186/s40478-026-02455-z

**Published:** 2026-10-28

**Authors:** Isil Keskin, Isabelle Sigfridsson, Ela Skagerberg Ekhtiari, Matthew Marklund, Samuel Mattson, Thomas Brännström, Stefan L. Marklund, Ulrika Nordström, Peter M. Andersen

**Affiliations:** 1 Department of Clinical Sciences, Neurosciences, Umeå University, 90185 Umeå, Sweden; 2 Department of Medical Biosciences, Umeå University, 90185 Umeå, Sweden

**Keywords:** Amyotrophic lateral sclerosis, ALS, SOD1, Templated aggregation, Strain morphology, Aggregate conformation, Sciatic nerve, Neurodegenerative disease, Motor neuron disease

## Abstract

Accumulation of misfolded SOD1 protein into intracellular aggregates, in the spinal cord (SpC) and brainstem, is a hallmark of amyotrophic lateral sclerosis caused by mutations in the *SOD1* gene (SOD1-ALS). Two distinct aggregate conformations, denoted strain A and strain B, were identified in the CNS of *SOD1*^*G85R*^ and *SOD1*^*D90A*^ transgenic (Tg) ALS mouse models, respectively. Both strains transmit templated SOD1 aggregation and ALS-like disease in Tg mouse models when inoculated into the SpC, whereas muscle or intraperitoneal inoculations, of up to 300 times more inoculate, did not. Here, we used strain A and strain B aggregate seeds, prepared from SpCs of the Tg mice, to investigate strain-specific differences in transmission of templated aggregation and ALS phenotype via a peripheral nerve. We show that both strains can transmit templated aggregation to the CNS via the sciatic nerve in *SOD1*^*G85R*^ Tg mice. Strain A resulted in shorter time to disease onset and earlier end-stage disease compared to strain B, and these strain-specific differences were accentuated in the sciatic nerve inoculation model compared to direct SpC inoculations. To further analyse the differences between strain A and strain B, we developed an immunohistochemical method that distinguishes the two SOD1 aggregate strains in tissue sections. This method revealed that strain B adopts an elongated, skein-like morphology, whereas strain A aggregates appear as accumulations of rounded, bead-like structures. These findings suggest that SOD1 aggregates forming in a peripheral nerve might transmit disease to the CNS and that different SOD1 aggregate strains can influence disease onset and progression. Our results warrant further exploratory studies of SOD1 aggregate conformations and their potential role in the manifestation of SOD1 mutant-specific disease phenotypes.

**Supplementary Information:**

The online version contains supplementary material available at https://doi.org/10.1186/s40478-026-02455-z.

## Introduction

Amyotrophic lateral sclerosis (ALS) is now recognized as a heterogeneous syndrome with respect to aetiology, clinical presentation, prognosis and neuropathology. The vast variability in ALS phenotypes is reflected in the multitude of genetic variants which may cause, or contribute to disease. More than 50 genes have been associated to ALS, and the most common are intronic hexanucleotide repeat expansions in *C9ORF72* and coding mutations in the *superoxide dismutase 1* (*SOD1*) gene. ALS-causing mutations in *SOD1* were first reported in 1993 [1]. Since then, 236 different *SOD1* mutations have been identified in ALS patients, and different mutations result in remarkably different disease characteristics: At one end of the clinical spectrum are patients heterozygous for the *A4V* or *G41S* mutations, which predominantly present with spinal motor neuron involvement, abrupt onset and rapid progression with less than two years survival time [2, 3]. At the other end are patients heterozygous for *G41D* or *L117V*, and those homozygous for *D90A*, which initially present with upper motor neuron symptoms, invariably have onset of paresis in the legs, and frequently live with ALS up to a decade or more [4, 5].

Mutations in *SOD1* typically destabilise the native protein structure, rendering cytotoxic misfolding and accumulation of protein inclusions. Such cytoplasmic inclusions of aggregated SOD1 are a hallmark of SOD1-ALS, both in patients [6, 7] and in transgenic (Tg) mouse models overexpressing ALS-causing human SOD1 variants [8–11]. Using a panel of short peptide antibodies specific to misfolded human SOD1 species, we have identified two structurally different strains of misfolded SOD1 aggregates in Tg mouse models. The unstable G85R mutant invariably forms strain A-aggregates in the CNS of *SOD1*^*G85R*^ Tg mice. In contrast, aggregates forming in early-onset Tg mice expressing the wild-type (wt) like human D90A variant predominantly adopt a different conformation, denoted strain B. Albeit, *SOD1*^*D90A*^ Tg mice that reach end-stage disease at old age contain a mix of A and B aggregates [12].

Several studies have provided compelling evidence for a prion-like propagation of SOD1 aggregation [13–19]. Our previous studies showed that both strain A and strain B SOD1 aggregate seeds, prepared from SpCs of terminally ill Tg ALS-model mice, induced spreading of template-directed SOD1 aggregation and premature fatal ALS-like disease upon inoculation into the SpCs of asymptomatic Tg mice expressing mutant SOD1. Additionally, inoculation of two seed preparations prepared from autopsy tissue from two *SOD1*^*D90A*^ homozygous patients, one transmitting strain A and the other strain B SOD1 aggregation, also induced premature ALS symptoms in Tg mice. These results indicate that both strain A and strain B can arise also in ALS patients. Importantly, the phenotype and progression varied depending on the propagated strain [18, 20].

We have further shown that muscle or intraperitoneal inoculations, of up to 300 times more strain A inoculate, failed to transmit disease to the CNS of *SOD1*^*G85R*^ Tg mice [21]. Notably, some organs, for example the liver and kidneys, contain similar or higher levels of the ubiquitously expressed SOD1 to those found in the CNS [22]. Still, we could neither detect transmitted aggregation nor any spontaneously arising aggregation in peripheral organs [21]. These results suggest that transmission of SOD1 aggregation from the periphery is an unlikely cause of ALS.

Studies of the *SOD1*^*G93A*^ Tg model have, however, showed that SOD1 aggregation appears early in the sciatic nerve and perturbs axonal transport before symptom onset [23, 24].

Several studies have further suggested that SOD1 aggregation can be transmitted to the CNS via peripheral nerves. This was studied by sciatic nerve inoculation in *SOD1*^*G85R-YFP*^ mice, using second-passage SpC homogenates from the same Tg mice, inoculated with in vitro-generated SOD1 aggregates, or SpC homogenates derived from Tg models or post-mortem tissue from patients with SOD1-ALS [17, 25, 26]. The *SOD1*^G85R-YFP^ construct enables visualization of the aggregation process, albeit the large YFP-tag could also alter aggregation propensity and conformation. Nonetheless, these studies support the hypothesis that pathogenic SOD1 aggregation causing ALS could originate in a peripheral nerve.

Here, we investigated two well-characterized, structurally distinct SOD1 aggregate strains that are relevant for human ALS and assessed their potential differences in transmission of SOD1 pathology to the CNS via a peripheral nerve. We inoculated either strain A or strain B seeds into the sciatic nerve of adult *SOD1*^*G85R*^ Tg mice. Both strains transmitted spreading premature SOD1 aggregation within the CNS and resulted in fatal ALS-like disease. Notably, the strain B seeds propagated SOD1 aggregation with strain B conformation in the recipient *SOD1*^*G85R*^ Tg mice, which inherently only develop strain A. Thus, we show here that strain-specific templated aggregation of SOD1 can be transmitted from a peripheral nerve to the CNS. Using immunohistochemistry (IHC) together with combinations of SOD1 peptide-antibodies, we can now distinguish between templated strain B and strain A aggregates and visualize their distinct morphologies in seed-inoculated mice. Our study highlights the need for further explorative studies of SOD1 aggregate strain conformations and their contribution to the phenotypic heterogeneity observed in SOD1‑ALS.

## Materials and methods

### Reagents and chemicals

Reagents and chemicals were obtained from Sigma-Aldrich or Thermo Fisher Scientific unless stated otherwise.

### Mouse husbandry and procedures

The Tg mice expressing human *SOD1*^*G85R*^ (line 148) [8] or *SOD1*^*D90A*^ [11] were backcrossed for more than 30 generations and maintained on the C57BL/6J background. In these colonies, the average lifespan of hemizygous *SOD1*^*G85R*^ mice is 398 ± 42 days (*n* = 86), whereas homozygous *SOD1*^*D90A*^ mice develop end-stage symptoms between 350 and 600 days. There is no significant difference between male and female mice within each line. SpCs from sacrificed, age-matched, non-Tg C57BL/6J mice from the breeding colony were used for preparation of control seeds. All animals were housed under environmentally controlled standard conditions with a 12 h light/dark cycle and free access to food and water. A plate of wet food was provided daily on the cage floor to facilitate free access after onset of paresis symptoms. The maintenance of the Tg mice and the experimental protocol were approved by the Umeå Regional Animal Research Ethical Board.

### Preparation of SOD1 strain A and strain B aggregate seeds

Strain A aggregate seeds were prepared from end-stage *SOD1*^*G85R*^ Tg mice (*n* = 3 + 3, two pooled preparations), and strain B seeds from *SOD1*^*D90A*^ Tg mice (*n* = 3) with early end-stage disease (384 ± 30 days). Both strain A and strain B aggregates can form in *SOD1*^*D90A*^ Tg mice, but B-aggregates are typically dominant in mice with short lifespans [12]. SpC homogenates from *SOD1*^*D90A*^ Tg mice were analysed by dot blot on filter captured aggregates and homogenates with high reactivity to the strain B specific Ab α111–127 were selected for seed preparation. Control seeds were prepared from age-matched non-Tg C57BL/6 mice (*n* = 3+3, two pooled preparations), essentially as described previously [18, 19, 21]. In summary, only proteinaceous components with molecular masses > ~ 5 × 10^6^ Da were sedimented and collected. Thus, the seed preparations will likely not contain monomeric, dimeric, or any small oligomeric SOD1 species.

We homogenized whole SpCs in 5 weight volumes (w/v) of ice-cold phosphate-buffered saline (PBS) containing 1.8 mM EDTA, 0.25 M guanidinium chloride (GdmCl), 2% (v/v) Nonidet P-40 (NP-40), and a Complete EDTA-free protease inhibitor cocktail (Roche Diagnostics) using an Ultraturrax apparatus (IKA) for 20 s, followed by sonication for 1 min. The homogenate was diluted with 0.66 volumes of water containing 1% NP-40 to achieve physiological ionic strength (0.15 M salt), sonicated for 1 min, and centrifuged at 1000 g for 20 min at 4 °C. The supernatants from three SpCs were pooled, supplemented with 3% iohexol and transferred to 4 ml UltraClear flexible ultracentrifugation tubes containing 0.25 ml (2 mm height) of 75.5% iohexol, followed by a layering of 1.5 ml (10 mm) of 13% iohexol, 1.5 ml (10 mm) of the homogenate containing 3% iohexol, and finally ≈ 0.75 ml PBS to fill the tubes to the top. The homogenate was then centrifuged through the iohexol density cushion for 2 h at 360,000 g at 4 °C and the suspension containing aggregated SOD1 and other heavy components that sedimented to the 13% / 75.5% iohexol interphase was collected. The iohexol was removed by dialysis against PBS (pH 7.0) and concentrated using a Spectra gel. For control and strain A seeds, two preparations, from three SpCs each, were pooled. The resulting preparations were aliquoted and stored at − 80 °C until used for inoculations. The concentration of SOD1 in the strain A and strain B seed preparations was assayed by western blot, including serial dilutions of a SOD1 standard, and estimated to ~ 3.8 ng/µl and ~ 4.6 ng/µl, respectively. The total protein contents of strain A (1559 ng/µl), strain B (986 ng/µl) and control seeds (1585 ng/µl) were estimated using Pierce BCA Protein Assay.

### Animal inoculations

Inoculations of aggregate seeds were performed in ~ 100-day-old asymptomatic *SOD1*^*G85R*^ Tg mice, anesthetized with 5% (v/v) isoflurane (Baxter), fixed on a small animal stereotactic frame (Kopf Instruments) and kept under anaesthesia with 1.5–2% (v/v) isoflurane. Eye protector gel was applied, and mice were injected subcutaneously with Carprofen (Rimadyl, 5 mg/kg, Pfizer) to relieve post-surgical pain. Body temperature was monitored with a rectal probe and regulated with a TCAT-2LV temperature controller (AgnTho’s).

For sciatic nerve inoculations, the lateral right hindlimb was shaved and disinfected before incision. The sciatic nerve was exposed between the biceps femoris and gluteus maximus muscles, carefully lifted and protected from drying with sterile NaCl. The epineurium was punctured using a fine needle. A separate 5 µl syringe with a 33-gauge needle (Hamilton) was used for each type of inoculum to prevent contamination. The needle was inserted ∼3 mm deep under the epineurium and reciprocated multiple times before inoculation, a procedure reported to enhance transmission efficiency of the infectious isoform of prion protein [27]. 1 µl of aggregate seed preparation was slowly injected under the perineurium over 3 min. The syringe was then slowly retracted over 3 min, the nerve was carefully repositioned between muscles, and the skin incision closed with stainless steel clips (Reflex Autoclip System, 10 mm, AgnTho’s). Surgical clips were removed two weeks after surgery.

SpC inoculations were performed as previously described [18, 19]. In summary, the recipient ~100-day-old asymptomatic *SOD1*^*G85R*^ Tg mice were anesthetized, and 1 µl of aggregate seed preparation was inoculated into the ventral horn of the left lumbar SpC between two vertebrae at the L2–L3 level. Injections were performed using a motorized micromanipulator, microinjection pump (AngTho’s AB) and a 5 µl syringe with a 33-gauge needle (Hamilton). Separate syringes were used for each type of inoculum to prevent contamination. The syringe was slowly retracted, the fascia sutured, and the skin incision was closed with Tissue Adhesive (3 M Vetbond).

After all surgeries, physiological saline was injected subcutaneously for rehydration, and mice were moved to a cage placed on a heating blanket for recovery before being returned to their home cage. After the procedure, mice were monitored to ensure full mobility and no signs of impairment.

### Monitoring of inoculated mice

All mice were weighed and examined for symptom onset weekly. In inoculated mice, the onset symptom was uniform hindlimb splaying of the ipsilateral hindlimb. In non-inoculated, control seed inoculated, and unsuccessfully inoculated mice (i.e. those that did not develop premature paresis), the onset symptoms varied between abnormal hindlimb splay or forelimb weakness. Unless otherwise stated, mice were sacrificed when considered terminally ill. The criterion for end-stage ALS-like disease was defined as advanced paralysis in both hindlimbs or more than 20% loss of body weight. In mice with prominent forelimb symptoms, end-stage criteria also included severe forelimb weakness, resulting in feeding difficulties or eye infection.

### Excluded mice

**Fig. 1:**
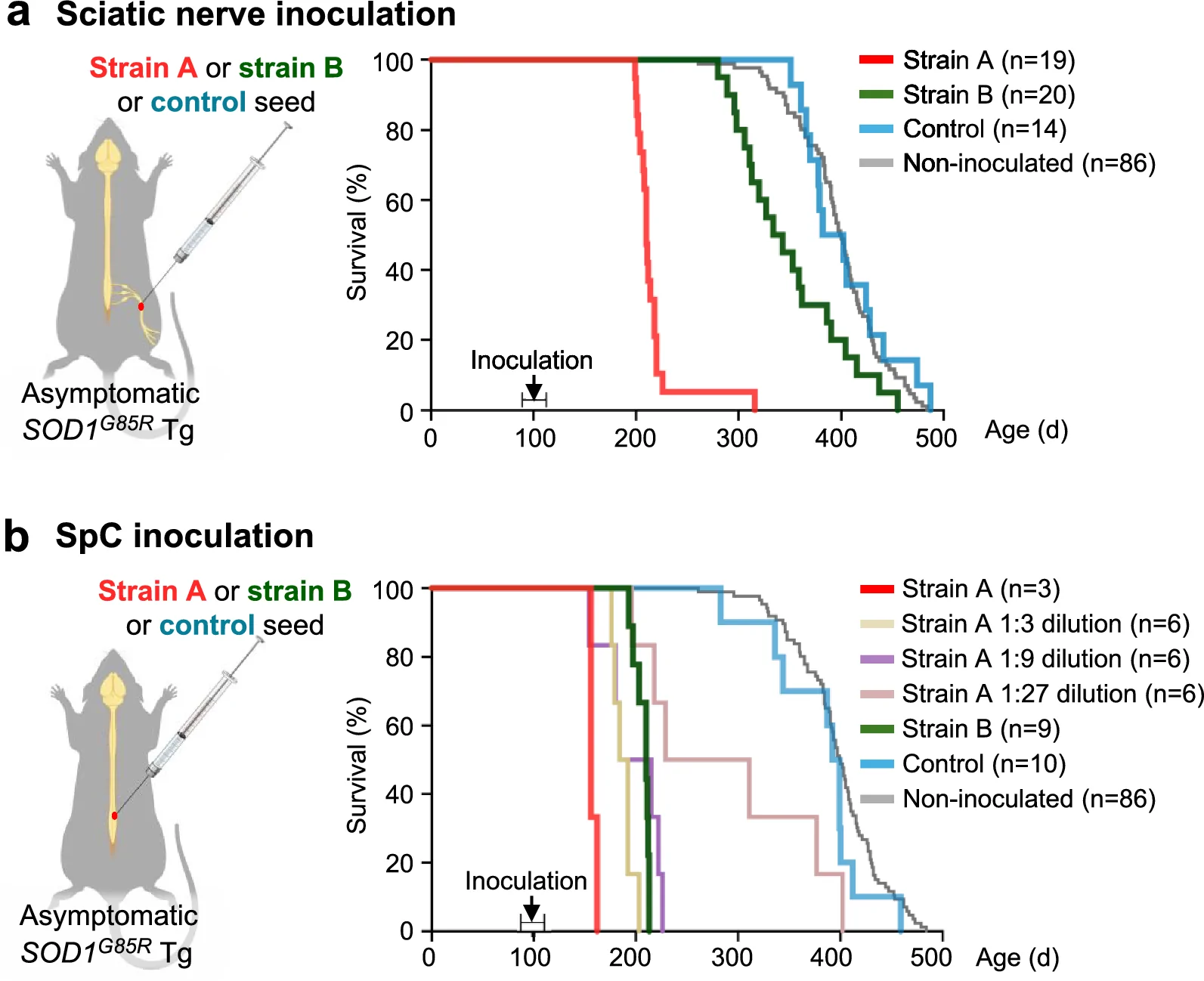
SOD1 aggregate strain and concentration-dependent effects on premature ALS-like disease. Asymptomatic, ~100-day-old, *SOD1*^*G85R*^ Tg mice were inoculated with 1 μl of strain A or strain B aggregate seed preparation from SpC tissue of end-stage paralytic *SOD1*^*G85R*^ and *SOD1*^*D90A*^ Tg mice, respectively, or control seeds from age-matched non-Tg C57BL/6 mice. Schematic images depict inoculation sites. Kaplan–Meier plots show the survival of seed inoculated, and non-inoculated *SOD1*^*G85R*^ Tg mice for reference. Black arrows indicate inoculation window. See Table 1 for detailed data and statistics. **a** Seed preparation was inoculated into the right sciatic nerve, and disease development was assessed until terminal paralytic disease. Both strain A and strain B seed inoculations resulted in premature disease development and significantly shorter survival compared to control inoculated mice (Table 1). Strain B inoculation resulted in significantly longer survival compared to strain A (*p* = 0.0001). **b** Aliquots of the same seed preparations were inoculated into the ventral horn of the lumbar SpC to assess the seeding activity in each preparation. Strain B inoculation resulted in significantly longer survival compared to undiluted strain A (*p* = 0.0003). Serial dilutions (1:3, 1:9 and 1:27) of strain A seed resulted in progressively longer survival time. Survival data for control seed SpC inoculations was obtained from Bidhendi et al. [18] and plotted for comparison. Schematic images created with BioRender.com

Four mice were excluded from the study and not reported in Results, Discussion, Figures, or Tables. Two mice that received sciatic nerve inoculation, one with strain A, and one with strain B seed, were euthanized 36 and 15 days after inoculation, respectively, due to poor healing of wounds adjacent to the injection site. One mouse that received sciatic nerve inoculation with a control seed was euthanized 85 days after inoculation due to non-surgery-related wounds. One strain A SpC-inoculated mouse was found dead, of unknown cause, 50 days after surgery. None of the excluded mice showed any paretic symptoms. Two out of 21 strain A sciatic nerve inoculated mice were excluded as outliers (> 3 SD from the average time to symptom onset) and were omitted from the survival curve in Fig. 1, and calculations in Table 1. Their long survival may have resulted from technical issues with the sciatic nerve inoculation protocol, as these inoculations, in contrast to the SpC inoculations, were performed with handheld syringes.

**Table 1:** Disease development in strain A-, strain B- or control-seed inoculated *SOD1*^*G85R*^ Tg mice

	Strain A		Strain B		Control	
	*n*	Days	*n*	Days	*n*	Days
***Sciatic nerve inoculations***						
Age at inoculation	19	108 ± 2^ ns^	20	103 ± 6^ ns^	14	105 ± 5
Time to symptom onset	19	90 ± 24****	20	211 ± 51***	14	277 ± 39
Duration of disease	19	18 ± 3^ ns^	20	35 ± 6****	14	21 ± 8
Time to fatal disease	19	108 ± 25****	20	246 ± 50**	14	298 ± 41
Age at fatal disease stage	19	216 ± 25****	20	349 ± 51**	14	403 ± 42
Weight loss from max. (%)	19	31 ± 7^ ns^	20	36 ± 7^ ns^	14	33 ± 8

	Strain A		1:3 Strain A		1:9 Strain A		1:27 Strain A		Strain B		Control	
	*n*	Days	*n*	Days	*n*	Days	*n*	Days	*n*	Days	*n*	Days
***SpC inoculations***												
Age at inoculation	3	95 ± 2^ ns^	6	106 ± 1^ ns^	6	107 ± 2^ ns^	6	105 ± 2^ ns^	9	101 ± 2^ ns^	10	98 ± 10
Time to symptom onset	3	45 ± 5**	6	55 ± 11***	5	71 ± 22***	6	112 ± 61***	9	47 ± 7****	10	238 ± 46
Duration of disease	3	18 ± 3^ ns^	6	25 ± 7^ ns^	5	25 ± 8^ ns^	6	70 ± 32^ ns^	9	59 ± 9^ ns^	10	45 ± 47
Time to fatal disease	3	63 ± 2**	6	80 ± 9***	6	88 ± 27***	6	182 ± 87*	9	106 ± 8****	10	284 ± 54
Age at fatal disease stage	3	158 ± 3**	6	186 ± 10***	6	195 ± 29***	6	287 ± 88*	9	207 ± 7****	10	381 ± 49
Weight loss from max. (%)	3	17 ± 5^ ns^	6	24 ± 8^ ns^	6	18 ± 6^ ns^	6	27 ± 9^ ns^	9	23 ± 5^ ns^	10	21 ± 12

Data are expressed as the mean ± SD, not significant (ns) *p* > 0.05; *p ≤ 0.05; **p ≤ 0.01; ***p ≤ 0.001; ****p ≤ 0.0001, analysed by Mann–Whitney U test comparing the effects of strain B, or stepwise dilutions of strain A, to the control seed preparation. (*n*) Number of inoculated and analysed mice. Time to symptom onset, and time to fatal disease are calculated from the day of inoculation

### Tissue collection

Mice were euthanized with a lethal intraperitoneal injection of pentobarbital (60 mg/mL, APL Pharma) and perfused transcardially with PBS. The SpC was retrieved by flushing with saline using a syringe inserted into the vertebral column, caudally to sacral levels. Brain stem and SpC were divided sagittally into right and left sides, and then into lumbar, thoracic, and cervical segments. Tissues from one sagittal half, alternating ipsi-, and contralateral sides, were snap-frozen in liquid nitrogen and stored at − 80 °C for biochemical analyses. Tissues from the other sagittal half were fixed in formalin and paraffin-embedded for IHC.

Mice selected for spatiotemporal, and confocal microscopy analysis of SOD1 pathology were sacrificed at 15, 30, and 60 days post-inoculation (p.i.), at symptom onset, and at end-stage disease (*n* = 3–6/time point) and perfused with PBS followed by formalin, and whole SpC dissected and processed for IHC analysis.

### Human SOD1 antibodies

The polyclonal rabbit SOD1 antibodies (rbAbs) raised against keyhole limpet hemocyanin-coupled peptides corresponding to amino acids (aa) 4–20, 24–39, 43–57, 57–72, 83–91, 86–94, 100–115, 111–127 and 131–153 in the human SOD1 sequence, were purified using Protein A-Sepharose (GE Healthcare) followed by Sulfolink gel coupled to the respective target peptides [7, 10]. A monoclonal mouse antibody targeting aa 143–153 (α143-153 mAb) in SOD1 was produced using standard hybridoma technique as previously described [28]. The α83-91 Ab (αStrainB^D90A^) does not bind G85R mutant SOD1, and the α86-94 Ab (αStrainB^G85R^), does not bind D90A mutant SOD1, due to the mutations in their respective target sequences. These antibodies were used to distinguish between strain B aggregates from *SOD1*^*D90A*^ Tg mice, used for strain B seed preparations, and templated strain B aggregates in the recipient *SOD1*^*G85R*^ Tg mice.

### Quantitative binary epitope-mapping (BEM) assay

**Fig. 2:**
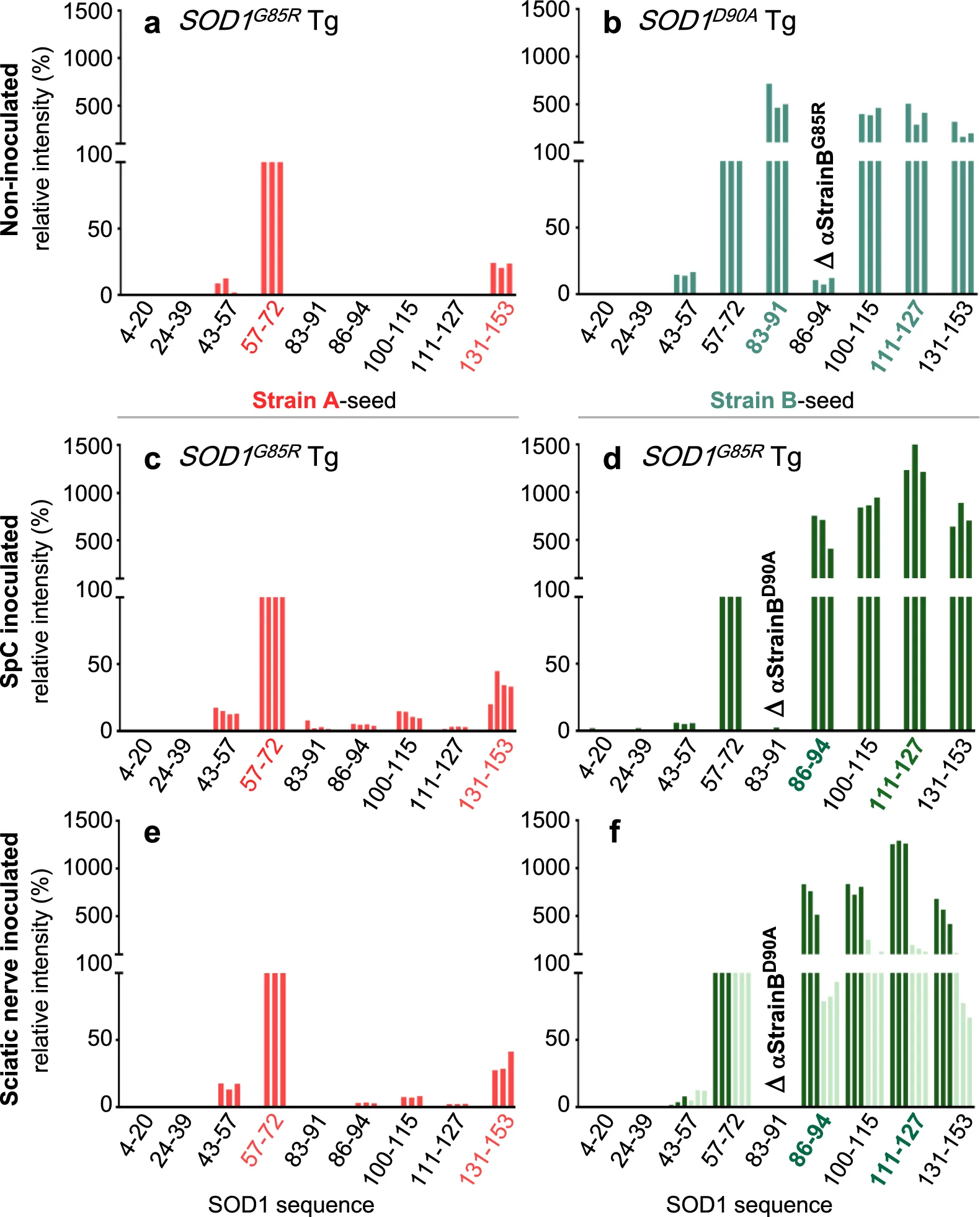
BEM patterns of seeds and aggregates formed in inoculated mice. Graphs show antibody reactivity patterns of filter-captured SOD1 aggregates from end-stage paralytic Tg mouse SpC homogenates. The x-axes represent the SOD1 protein sequence, highlighting the peptides aa 4–20, 24–39, 43–57, 57–72, 83–91, 86–94, 100–115, 111–127 and 131–153 used to raise the nine polyclonal rbAbs. The staining intensities were normalized against α57-72 Ab (set to 100%) to facilitate the comparison of patterns. Note the split y-axes used to visualize low Ab reactivities. **a** Typical strain A patterns detected in non-inoculated *SOD1*^*G85R*^ Tg mice (*n* = 3) used for strain A seed preparation. **b** Representative strain B patterns detected in non-inoculated *SOD1*^*D90A*^ Tg mice (*n* = 3), used for strain B seed preparations. Note that the α83–91 (αStrainB^D90A^) Ab and the α86-94 (αStrainB^G85R^) Ab can distinguish between strain B aggregates formed by the SOD1^D90A^ mutant protein in the donor mice, and templated SOD1^G85R^ strain B aggregates formed in the recipient mice, as their target epitopes include aa G85 and aa D90, respectively. **c** BEM reactivity patterns of aggregates formed in SpC inoculated mice, using the strain A seed preparation (*n* = 4) or **d** the strain B seed preparation (*n* = 3). **e** Strain A sciatic nerve inoculated mice show a typical strain A pattern (*n* = 3). **f** Strain B inoculation in the sciatic nerve (*n* = 6 mice) resulted in typical strain B patterns (*n* = 3, dark green bars), or a pattern suggesting development of co-existing strain A and strain B aggregates (n=3, light green bars)

Human SOD1 aggregate contents in the CNS tissues of seed inoculated *SOD1*^*G85R*^ Tg mice were quantified using a binary epitope-mapping (BEM) assay essentially as previously described [12]. Tissue homogenates were prepared from the lumbar, thoracic, and cervical SpC, and the brainstem. We used 25 weight volumes of ice-cold PBS containing 1.8 mM EDTA, 1 mM DTT, and a Complete EDTA-free protease inhibitor cocktail (Roche Diagnostics) using an Ultraturrax apparatus (IKA) for 20 s, followed by sonication for 1 min. The resulting crude tissue homogenates were stored at − 80 °C for the subsequent analysis of SOD1 aggregate contents. For the BEM assay, the tissue homogenates were further diluted in 20 volumes of PBS containing 1 mM DTT, 1.8 mM EDTA, and 1% (v/v) NP-40, sonicated for 30 s and centrifuged at 1000 g for 10 min at 4 °C. The resulting supernatants were serially diluted 1:1 in PBS and SOD1 aggregates were captured on 0.2 µm cellulose acetate filters using a 96-well dot-blot apparatus (Whatman GmbH). For the BEM assay, filters were cut into strips, blocked with 5% (w/v) dry milk in TBS containing 0.1% (v/v) Tween 20 for 1 h at room temperature, and incubated with antibodies in the blocking solution overnight at 4 °C. We used the primary rbAbs targeting aa 4–20, 24–39, 43–57, 57–72, 83–91, 86–94, 100–115, 111–127 and 131–153 of the human SOD1 protein sequence. The α57-72 Ab and α131-153 Ab show reactivity with both strain A and B aggregate conformations. In contrast, the 83–127 sequences of SOD1 are unable to interact with the antibodies targeting these sequences in the strain A conformation, but avidly reactive in the strain B conformation. Additionally, we used the α83-91 Ab (αStrainB^D90A^), which do not bind G85R mutant SOD1, and the α86-94 Ab (αStrainB^G85R^), which do not bind D90A mutant SOD1, to distinguish between strain B aggregates from *SOD1*^*D90A*^ Tg mice, used for strain B seed preparations, or templated strain B aggregates in the recipient *SOD1*^*G85R*^ Tg mice (see Fig. 2). The immunoreactivity was detected similarly to western blotting using ECL Select reagent (GE Healthcare), recorded on a ChemiDoc Touch Imaging System (BioRad), and analysed using Image Lab software (BioRad). As standard for quantifications, we used the α57-72 Ab reactivity (set to 1) to a SpC homogenate from an end-stage *SOD1*^*G93A*^ Tg mouse, which was included on all filters.

### Immunofluorescent histochemistry and microscopy

Paraffin-embedded SpC tissues, individual, or tissue microarray blocks generated with the TMA Grand Master (3DHISTECH), were cut with a microtome (Microm HM400). The 5 μm thick sections were mounted on glass slides, deparaffinized, rehydrated, and then exposed to a 4 min pre-treatment with Ventana Protease 1 solution using the Ventana BenchMark Ultra (Ventana Medical Systems). The advantage of this short endonuclease treatment is that monomeric disordered SOD1 is partly degraded, which allows improved visualization of aggregated SOD1 species using fluorescent IHC. Sections were treated with blocking solution, containing 10% (v/v) goat serum in Tris-buffered saline (TBS) and 0.3% (v/v) Triton X-100, for 1 h at room temperature, before incubation with primary antibodies in TBS containing 5% (v/v) goat serum and 0.1% (v/v) Triton X-100 overnight at 4 °C. We used mAb α143–153 (1 μg/ml) and the rbAb αStrainB^G85R^ (8.6 μg/ml), described above. Sections were washed with TBS containing 0.1% (v/v) Triton X-100 and incubated for 2 h at room temperature with Alexa-Fluor conjugated secondary antibodies (1:1000, Invitrogen) and 4′,6-diamidino-2-phenylindole (DAPI; 0.3 μM) for nuclear counterstaining. Following washing with TBS containing 0.1% (v/v) Triton X-100, sections were mounted with a Fluorescence Mounting Media (Dako).

For immunolabelling of seed preparations, we diluted 2 μl of the individual preparations in 20 μl PBS. The diluted seeds were then placed on Superfrost™ Plus Microscope Slides and spread out over a ~ 1 cm^2^ surface and allowed to semidry in RT to allow surface attachment, before processed with fluorescent IHC as described above, protease treatment excluded.

Fluorescence images were obtained using an Olympus BX 61 microscope equipped with cellSens Standard imaging software. Immunolabeled sections were further examined with confocal microscopy. Z-stacks were collected using either a Nikon A1 microscope, NIS-elements software and a 40× water immersion objective, or a Zeiss 710 confocal microscope. using the 63×/1.4 oil objective and the ZEN software. All images were processed and analysed with ImageJ software.

### Quantification of SOD1 aggregates in the ventral horn of the SpC

Formalin-fixed, paraffin-embedded tissue sections of alternating ipsilateral or contralateral ventral horns were double-labelled with mAb α143–153 and αStrainB^G85R^, as described above, while the corresponding sagittal halves were processed for BEM analysis. Images of the ventral horn were acquired at cervical, thoracic and two lumbar levels (3 mm apart), using a 40× magnification objective, and identical camera settings for the same antibody in all sections. Images were processed and analysed with ImageJ software. The three channels in each image were then segmented into a binary image of labelled aggregates and background using contrast-enhancement, fluorescence intensity thresholding, and size exclusion-filtering using the ImageJ software. Strain B aggregates were defined by colocalization of the mAb α143–153 and αStrainB^G85R^ Ab, and identified by creating a mask selecting only co-localized pixel objects (ROIs) above the threshold level, using the colocalization-plugin [29]. Strain A aggregates were counted by identification of pixel areas labelled by mAb α143–153 alone and localized at a minimum of 10 pixels distance away from a co-localized area (10 sequential 1-pixel dilations of colocalized ROIs and exclusion of the resulting area in the mAb α143–153 alone ROI identification). The reason for this was to avoid duplicate counting caused by the imperfect overlap of staining intensity threshold areas for each antibody. The steps described above were combined into a macro used for all images.

### Spatiotemporal analysis of spreading SOD1 aggregation

Formalin-fixed, paraffin-embedded tissue sections were fluorescently double-labelled, as described above, using mAb α143–153 and αStrainB^G85R^ rbAb. The ipsilateral and contralateral sides of whole lumbar, thoracic and cervical SpC sections were examined for spatiotemporal distribution and amount of SOD1 aggregates. The regions scored were the ventral horn, the dorsal horn, and the central grey matter, referred to in this report as pericentral grey matter. This region was delimited by a coronal plane at the grey-white matter junctions of the anterior white commissure and posterior white commissure. The three regions were scored based on aggregate abundance. No objective quantitative method was applied, aggregates were visually graded by ocular scoring, using a 20× objective and Olympus BX 61 microscope; from widespread (+ + +), (+ +), ( +), to no aggregation (–) in the respective regions. To show the average results from 3–4 scored mice at each time point, we translated the estimations into numerical values (0–3).

### Statistical analyses

Statistical analyses were performed using Prism (GraphPad Software). Differences between two groups (Table 1) were assessed using the Mann–Whitney U test. A *p* value ≤ 0.05 was considered statistically significant. Data are presented as mean ± standard deviation (SD).

Differences between survival curves (Fig. 1) were analysed using the Mantel-Cox test.

**Fig. 3:**
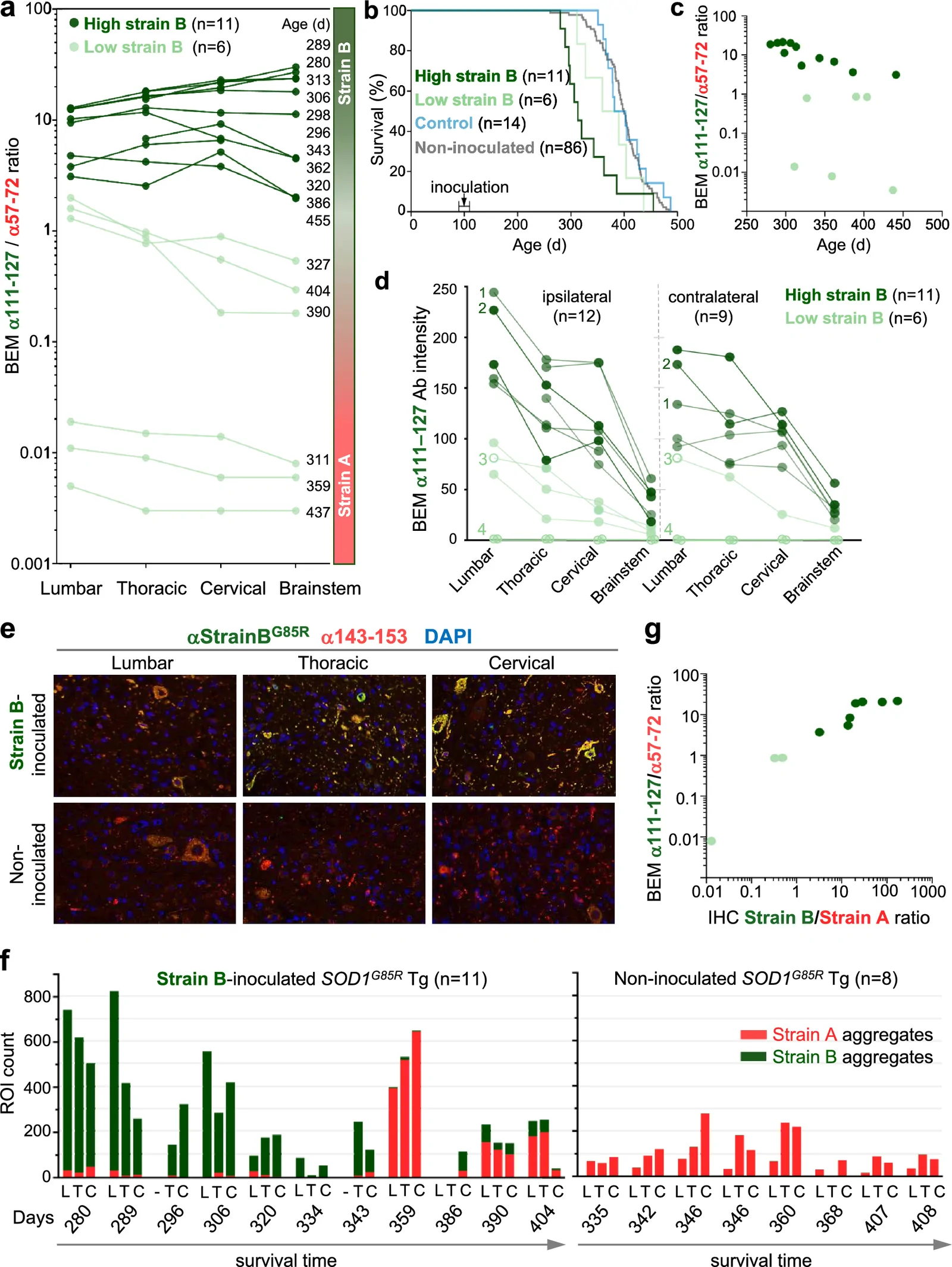
Estimations of templated strain B aggregation in sciatic nerve inoculated mice. **a** Relative ratios of strain B versus strain A aggregate levels in lumbar, thoracic, and cervical SpC, and brainstem of end-stage strain B-inoculated *SOD1*^*G85R*^ Tg mice. Y-axes represent the ratio of α111–127 versus α57–72 Ab intensities, the two antibodies with the highest intensities in strain B and strain A, respectively. High strain B levels are represented in (dark green). Low strain B ratio in (light green). Age (days) at end-stage disease is given for each analysed mouse in the right column. **b** Kaplan Meier plot shows survival of strain B inoculated mice stratified according to high or low strain B ratio. **c** Plot shows significant negative correlation between the mean of all α111–127/α57–72 intensity ratios, from spinal cord and brainstem, and survival time (*r*_*s*(16)_= − 0.7108, *p* < 0.01). **d** Distribution of strain B aggregates in the ipsilateral and contralateral sides along the neuroaxis. There was no significant difference between the two sides (*n* = 17 mice). Both sagittal halves were analysed in (*n* = 4) mice, indicated 1–4 in the graph. Circle represent data point generated from a pool of ipsilateral and contralateral lumbar tissue. **e** Representative immunofluorescent images used for IHC quantification of strain A and strain B aggregates in the ventral horn of end-stage disease strain B inoculated mice. **f** Graph shows estimated strain A and B aggregate load (object count) in lumbar (L) (mean of *n* = 2 sections/mouse), thoracic (T) and cervical (C) levels (*n* = 1 section/mouse), (–) No tissue available. Strain B aggregates (green) were defined as colocalization of the αStrainB^G85R^ rbAb and the α143–153 mAb, and strain A as α143–153 mAb labelling alone (red). X-axes represent age at end-stage disease. **g** Plot shows significant correlation (*r*_s(9)_=0.9879, *p* < 0.0001) between the mean strain B/strain A ratio estimated with BEM, and the IHC ROI count analysis methods. **a**–**g** See detailed description of available or missing tissues for each analysis in Table S1

Correlation analysis was performed using the non-parametric Spearman rank correlation coefficient (Figs. 3c, g and S1).

## Results

### Both strain A and B SOD1 aggregate seeds can transmit premature ALS-like disease via the sciatic nerve

Strain A, strain B, and control seeds were prepared by density gradient ultracentrifugation of SpC homogenates from end-stage Tg mice expressing the constitutively misfolded *SOD1*^*G85R*^ or the wt-like *SOD1*^*D90A*^, and age-matched non-Tg C57BL/6, respectively. The *SOD1*^*D90A*^ SpC homogenates used were analysed with dot blot on filter-captured aggregates using α57–72, and α111–127 Abs to confirm high level strain B conformation before seed preparation (see BEM analysis below for details).

We inoculated 1 µl of either seed preparation into the sciatic nerve of ~ 100-day-old *SOD1*^*G85R*^ Tg mice. This allows for a ~ 200-day symptom-free window to study seeded aggregation and premature disease onset before inherent accumulation of strain A aggregates and ALS-like disease develops due to the *SOD1*^*G85R*^ Tg overexpression. Both strain A and strain B induced premature ALS-like symptoms and significantly shortened survival. In contrast, there was no significant difference in mean survival between mice injected with control seed and non-inoculated mice (Fig. 1a and Table 1). Notably, the sciatic nerve inoculations resulted in longer time to onset and end-stage disease compared to our previously reported results, using the SpC inoculation model [18].

### Reduced propagation efficacy in the sciatic nerve inoculation model compared to direct SpC inoculations

We hypothesized that either our new seed preparations contained low seeding activity or that the seeding activity reaching the CNS after sciatic nerve inoculation was reduced due to limitations in axonal uptake and retrograde transport of propagated seeds. To test this, we inoculated 1 µl of either of the same undiluted seed preparations into the lumbar SpC of asymptomatic, ~ 100-day-old *SOD1*^*G85R*^ Tg mice. In line with our previous results, both the strain A and strain B seed preparations induced premature ALS-like disease, and both current seed preparations had a somewhat higher seeding activity than those used in our previous studies (Fig. 1b and Table 1) [18]. To investigate how limited amounts of seeding activity reaching the CNS delays disease development, we next performed a dose–response study using the same strain A seed preparation, where three- (0.33×), nine-, (0.11×) or 27-fold (0.04×) dilutions were inoculated into the SpC. Higher dilutions resulted in progressively prolonged mean survival time, and 27-fold dilution resulted in mean onset and survival time exceeding that of the sciatic nerve strain A inoculated mice (Fig. 1b and Table 1). Thus, we conclude that the sciatic nerve inoculation model is less efficient than direct SpC inoculations.

### Structural characterization of templated SOD1 aggregation

To investigate if sciatic nerve seed inoculation transmits template-directed aggregation, we analysed the conformation of SOD1 aggregates in the SpCs of seed-inoculated mice that reached end-stage disease. We used a modified BEM analysis based on a panel of nine polyclonal rbAbs raised against small peptides, collectively covering 90% of the SOD1 amino acid sequence. The α57–72 Ab and α131–153 Ab show reactivity with both strain A and strain B aggregate conformations. The aa 83–127 sequences are incorporated in the tightly ordered core of strain A aggregates and do not interact with antibodies, but are avidly reactive in the strain B conformer, in which the entire C-terminal half seems to protrude into the solvent in a disordered manner [12] (Fig. 2a, b). Additionally, the antibodies targeting aa 83–91 (αStrainB^D90A^) and aa 86–94 (αStrainB^G85R^) are unable to bind the G85R, or D90A mutant protein, respectively, due to mutated residues in their target epitopes. Thus, these antibodies can differentiate between strain B aggregates from *SOD1*^*D90A*^ Tg mice in the seed preparations, and templated strain B aggregates in the recipient *SOD1*^*G85R*^ Tg mice (Fig. 2b, d, f).

The strain A seed induced premature aggregation with a typical strain A pattern in both the SpC and the sciatic nerve inoculation model (Fig. 2c, e). Strain B induced strain B aggregation in the SpC inoculation model (Fig. 2d). In contrast, while some strain B sciatic nerve inoculated mice showed a typical strain B pattern, indicating efficient propagation of template-directed strain B conformation formed by the G85R mutant protein in the recipient mice, others showed reduced intensities in the strain B-specific aa 83–127 domains (Fig. 2f). We noted that the mice that did not develop typical strain B patterns in the BEM analysis generally had longer survival times (mean 368 days). At this time, the spontaneous age-dependent strain A aggregation, typically detected with BEM analysis in 300–400 days old non-inoculated *SOD1*^*G85R*^ Tg mice, would likely emerge [18], suggesting that these atypical BEM patterns represent a mix of strain A and strain B aggregates.

To analyse this, we estimated the relative proportion of strain B versus strain A using the intensity ratio between the α111–127 Ab, which is specific, and shows the highest reactivity to strain B, versus the α57–72 Ab, which shows the highest reactivity to strain A (Fig. 2) [12]. The ratio analysis of the strain B inoculated mice suggests different proportions of co-existing strain A and B. Mice with a higher strain B ratio at lumbar levels typically also had the highest ratio at rostral levels, indicating efficient propagation of templated aggregation along the neuroaxis. In contrast, those with low lumbar ratio typically showed decreasing ratios in cervical, and brain stem tissue homogenates (Fig. 3a). Indeed, mice with the highest strain B ratio generally had shorter lifespans compared to mice with a mix of strain A and B aggregates (Fig. 3b), and there was an overall significant negative correlation between the lifespans and mean of the strain B ratios shown in Fig. 3a (Fig. 3c). We found no significant differences in strain B aggregate load between the ipsilateral and contralateral sides (Fig. 3d).

### Immunohistological analysis of templated strain A and strain B SOD1 aggregates

Next, we aimed to analyse the different proportions of co-existence of strain A and strain B using immunofluorescent labelling in tissue sections and strain-specific combinations of SOD1 peptide antibodies. We identified a combination of antibodies which distinguish between strain A and strain B aggregates, using the αStrainB^G85R^ rbAb (aa 86–94), which only recognizes strain B in the BEM analysis, in combination with the α143–153 mAb which binds to both strains. Strain A or strain B inoculated *SOD1*^*G85R*^ Tg mice that reached end stage at different ages were analysed, and age-matched asymptomatic, or end-stage non-inoculated *SOD1*^*G85R*^ Tg mice were used as controls.

Strain A inoculated mice displayed premature SOD1 pathology labelled with α143–153 mAb, whereas co-labelling of the αStrainB^G85R^ and α143–153 Abs was only found in strain B-inoculated mice (Fig. 3e). The analysis of aggregation assemblies in the SpC tissue from end-stage strain B inoculated animals further showed that some strain B inoculated mice contained a mix of strain A and B aggregates, supporting our estimations using the relative ratios in the BEM analysis. We next performed a quantitative analysis of fluorescently labelled aggregates in the lumbar, thoracic, and cervical ventral horns of end-stage disease mice and found a significant negative correlation between the mean αStrainB^G85R^/α143-153 positive ROI count and increasing age at end stage (*n* = 11, *p* < 0.01, Figs. 3f and S1). This confirmed that mice with faster disease progression developed higher levels of templated strain B aggregates. We next compared these results with the α111–127 /α57–72 Ab ratios, in the individual mice analysed using both methods. The results from the two methods, using two different sets of antibodies targeting distinct sequences of misfolded SOD1, showed a strong significant correlation (*n* = 10, *p* < 0.0001, Fig. 3g and Table S1).

**Fig. 4:**
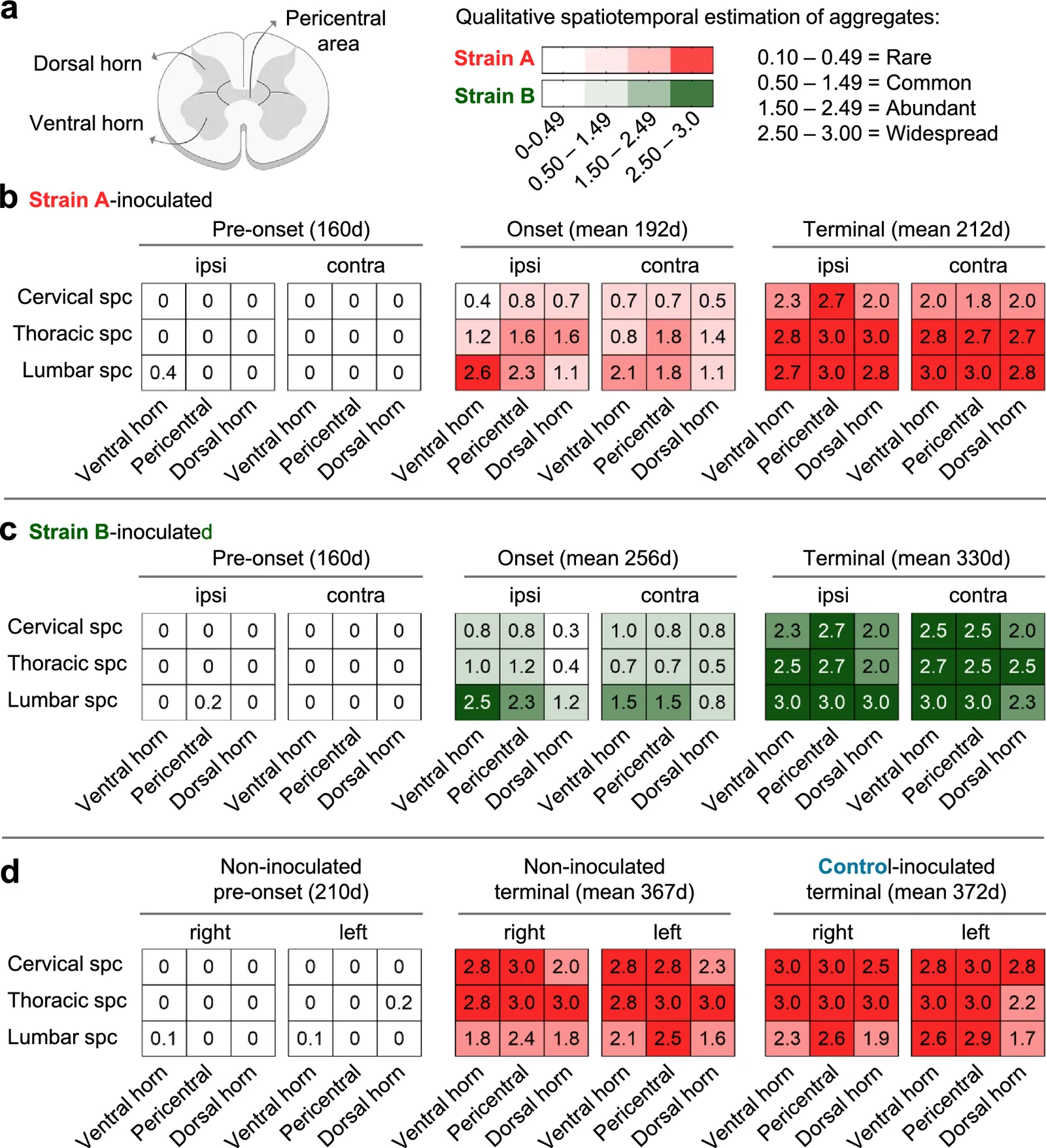
Progressive spread of strain A and strain B aggregate pathology. Templated aggregation was estimated using immunofluorescence and αStrainB^G85R^ Ab to identify strain B, and α143–153 mAb alone to identify strain A. **a** SOD1 aggregation pathology was assessed in 3 regions of the SpC; dorsal horn, ventral horn and pericentral area in sections from lumbar (mean of *n* = 2/mouse), cervical (*n* = 1/mouse) and thoracic (*n* = 1/mouse) levels. Different levels of aggregation pathology are shown using colour shades and numeric representations. **b**, **c** Rare aggregates were first observed in the ipsilateral lumbar SpC at 30 days p.i. for strain A inoculated mice (*n* = 4/time point) (data not shown), and at 60 days p.i. for strain B inoculated mice (*n* = 3/time point). At symptom onset, templated aggregation was detected at all levels of the SpC, but most abundant in the ipsilateral lumbar ventral horn and in the pericentral area. At end-stage, widespread strain A and strain B templated aggregation had propagated to all levels of the SpC, with no ipsi- to contralateral difference. **d** Age-matched, ~ 200-day-old asymptomatic, non-inoculated *SOD1*^*G85R*^ Tg mice (*n* = 6) contained minute amounts of α143-153 positive strain A aggregation. At end-stage disease, aggregates were most abundant in the cervical SpC in both control seed inoculated (*n *= 3) and non-inoculated mice (*n* = 3)

We also performed a qualitative spatiotemporal analysis based on the regional abundance of SOD1 pathology. This indicated that both strain A and strain B aggregation pathology was initiated in the ipsilateral ventral horn of the lumbar SpC and propagated to the contralateral side and rostrally via the pericentral area (Figs. 4, and S2). Thus, we did not find any clear differences in the spreading pattern between the two strains.

### Distinct morphologies of strain A and strain B SOD1 aggregates

**Fig. 5:**
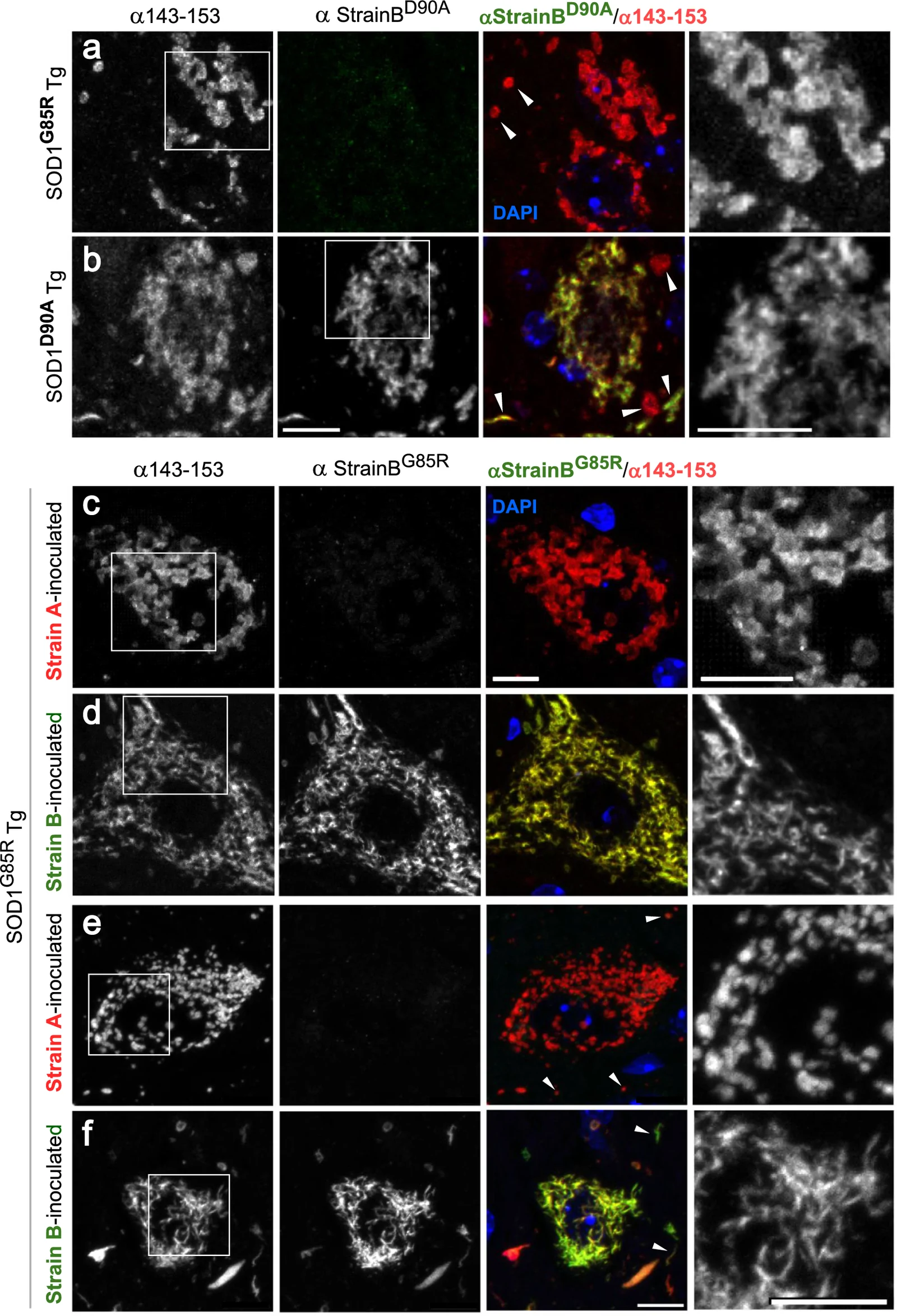
Morphological distinction between strain A and strain B SOD1 aggregates. Images are confocal MAX projections of strain A and strain B SOD1 assemblies in lumbar motor neurons at end stage disease. Co-labelling of α143-153 mAb and αStrainB^D90A^, or αStrainB^G85R^ rbAbs were used to detect strain B, and α143-153 mAb labelling alone to identify strain A SOD1 aggregates. **a** Non-inoculated *SOD1*^*G85R*^ Tg mice, used for strain A seed preparation, develop rounded amorphous grain-like aggregates. **b** Non-inoculated *SOD1*^*D90A*^ Tg mice can develop both skein-like elongated strain B assemblies and rounded strain A aggregates. SpC homogenates with predominately strain B aggregations were used for strain B seed preparation. **c–e** Strain A inoculated *SOD1*^*G85R*^ Tg mice develop premature strain A aggregation of similar morphology as the donor mice. **d–f** Strain B inoculated *SOD1*^*G85R*^ Tg mice develop the elongated strain B aggregate morphology, normally only detected in the *SOD1*^*D90A*^ Tg donor mice. White arrowheads indicate strain specific aggregation in the neuropil. Scale bars represent 10μm

During the IHC analysis of the seed inoculated mice, we noted a distinct morphological difference between the two SOD1 aggregate strains. To investigate this further we performed the same mild protease pre-treatment and IHC protocol on SpC tissue sections from end stage disease, non-inoculated *SOD1*^*G85R*^ and *SOD1*^*D90A*^ Tg mice, using the α143-153 mAb in combination with αStrainB^G85R^, or αStrainB^D90A^ rbAb, respectively. Confocal imaging analysis showed that *SOD1*^*G85R*^ Tg mice form grain-like, rounded strain A aggregations (Fig. 5a). In contrast, *SOD1*^*D90A*^ Tg mice form elongated, skein-like strain B assemblies in the cytoplasm of motor neurons and in the neuropil, but also co-existing rounded strain A aggregates (Fig. 5). Confocal analysis of the strain A, and strain B seed inoculated *SOD1*^*G85R*^ Tg mice showed that these strain specific morphologies were templated to the CNS via the sciatic nerve in the recipient mice (Fig. 5c–f).

We further sought to investigate if we could detect these morphological differences in the strain A and strain B seed preparations. Control, strain A and strain B seeds were fluorescently labelled with the antibodies to detect SOD1 positive assemblies in the preparations. However, using this method, we were unable to detect any apparent differences between immunoreactive particles in the two seed preparations. No SOD1 immunoreactivity was detected in the control preparation (Fig. S3).

In summary, strain A and strain B aggregate pathology can be distinguished and analysed in tissue sections using IHC labelling. The BEM analysis provides the opportunity to assess more epitopes, using a battery of Abs covering 90% of the SOD1 sequence to analyse the conformation of the SOD1 aggregates, but the resulting reactivities are a summary of any mixed conformers in the analysed tissue homogenates. Moreover, using this method, we have not succeeded to extract sufficient amounts of aggregates to characterise the conformation of SOD1 aggregates in patient tissues. Future refining of IHC methods, using multiplexed short-peptide SOD1 antibodies, might reveal intermediate, or other SOD1 conformers in Tg mouse models and help characterize the SOD1 aggregates found in the few remaining motor neurons in patients at end-stage disease.

## Discussion 

We have previously shown that the microenvironment influences SOD1 aggregate conformation, and that the strain A and B which arise in the CNS of Tg mice differ from aggregate conformers that form in vitro and in transfected cell lines [30]. Here, we show that two CNS-relevant strains can transmit templated SOD1 aggregation and premature ALS-like disease when inoculated into a peripheral nerve. Xu et al. recently suggested that the propagation rates of aggregated SOD1 variants associated with slow- or fast-progressing ALS reflect the clinical progression rates seen in patients [26]. In our study, inoculation with strain B, forming in homozygous *SOD1*^*D90A*^ Tg mice, resulted in a significantly reduced propagation rate compared to strain A.

Protein aggregation is, however, subtly related to amino acid composition [31, 32] and we have previously demonstrated that SOD1 aggregate structure is influenced by single amino acid exchanges in disease-associated mutant variants [30]. Consequently, the *SOD1*^*G85R*^ which normally forms strain A in the recipient mice will likely exhibit higher resistance to adopt the strain B conformation. Such intrinsic restraint in adapting to conformers with propagation capacity could contribute to strain-specific disease courses. Thus, the differences in propagation rate might be the result of heterologous versus isologous conformation in the inoculated seed preparations [33]. Further inoculation studies, using *SOD1*^*D90A*^ Tg mice as recipients, will be important to understand if the differences in strain A and strain B propagation rates are specific to the *SOD1*^*G85R*^ inoculation models.

Using either inoculation model, the sciatic nerve or the SpC, the seeds will primarily be deposited in the extracellular space where movement of protein assemblies are restricted, allowing prolonged local contacts for potential cellular uptake [34, 35]. Still, the extracellular space is in a continuum with the CSF that moves in alternating directions within the CNS and might contribute to transmission of seeded aggregation along the neuroaxis [36]. In the sciatic nerve, extracellular aggregates would likely be hindered from entering the CNS at the root attachment zones [37]. Moreover, the CSF flows in an essentially anterograde direction and will likely contribute to removal of infectious aggregates from the peripheral nervous system. Consequently, any transmission of infectious aggregates from peripheral nerves to the CNS should therefore result from axonal uptake and high jacking of the axonal transport systems. The dynein motor complex might be responsible for the retrograde transport of infectious SOD1 aggregates [38] and operates at rates of the order of mm/h [39, 40], meaning that transported aggregates could reach the SpC within a day post inoculation. Hence, the delayed symptom onset in the sciatic nerve inoculation model suggests that only a fraction of the inoculated seeding activity is propagated to the CNS. These results show that the SpC inoculation model is probably better suited for inoculation studies of patient-derived material, which likely contains lower seeding activity compared to overexpressing Tg mouse tissues.

In the rat, motor axons account for less than 10% of the axons in the sciatic nerve, and the remainder are mainly sensory axons [41]. Assuming that the nerve composition is comparable in mice, the potential for transmission to dorsal sensory neurons is prevalent. In line with this, Ayers et al. reported onset of premature SOD1 aggregation in the ipsilateral dorsal root ganglion in sciatic nerve inoculated *SOD1*^*G85R−YFP*^ Tg mice, followed by aggregation in the dorsal horn [17]. Here, we focus on the SpC and find onset of aggregation in the ventral horn. Although we find aggregations in the dorsal horn at symptom onset, the largest amounts of both strain A and B aggregates are found in the ipsilateral lumbar ventral horn (Figs. 4 and S2). This suggests that uptake and/or transmission via motor axons is higher, or that motor areas are more susceptible to SOD1 aggregation. In contrast to Ayers et al. [17], we used Tg mice expressing non-tagged SOD1^G85R^ protein. A large YFP-tag likely stabilizes the otherwise highly unstable SOD1^G85R^ mutant protein and might increase the levels of accumulated misfolded protein that in turn would allow seeding in dorsal neurons already at an early stage. Other explanations could be increased aggregation propensity imposed by the YFP-tag, or differences in expression levels in various cell types depending on the insertion site of the Tg construct.

As the disease advances, SOD1 aggregation propagates to the contralateral side, comprising the ventral horn as well as pericentral and dorsal regions, and bilaterally extends rostrally until it ultimately encompasses the entire SpC. This likely results from a combination of transfer within connected neural networks and cell-to-cell transfer [39]. Although sensory involvement has been reported in some SOD1-ALS cases [4, 42, 43], this widespread aggregation is different from the typical motor area preponderance seen in patients and is likely related to the high SOD1 overexpression required for disease development within the short lifespan of the Tg mouse models [44].

Compared with the Spc inoculation model, the symptoms showed a distinct ipsilateral to contralateral progression, with severe paralysis in both hindlimbs before notable forelimb symptoms developed. This, in combination with early accumulation of aggregations in the pericentral area, might suggest that commissural interneurons, crossing the SpC midline, efficiently transmit templated aggregation, not only to the contralateral motor area but also via rostrocaudal projections on both sides. This spreading pattern might indicate that the transmission is initially predominantly driven via local synaptic connections of a few infected neurons. Conversely, in the SpC inoculation model, a multitude of cells with connectivity in the neuropil surrounding the inoculation needle trajectory are likely infected shortly after inoculation.

We also visualized the unique structural features of strain A and strain B SOD1 aggregates in both donor and recipient mice. While strain A forms dense rounded beads, strain B forms skein-like elongated assemblies in both non-inoculated *SOD1*^*D90A*^, and in strain B inoculated *SOD1*^*G85R*^ Tg mice. Indeed, morphological differences of SOD1 aggregates have been described in post-mortem tissue from ALS patients expressing different SOD1 mutants [7, 45], but further studies are needed to understand their impact on disease phenotype and if they can be stratified into distinct strains in patients.

## Conclusions

Our study suggests that pathogenic SOD1 aggregation may not only originate in the CNS but also in peripheral nerves, which has been corroborated by earlier reports. Here we expand the current knowledge by showing that two strain-specific conformations, which are relevant to human ALS, and their distinct morphologies can be transmitted to the CNS via a peripheral nerve. Our results also suggest that potential variations of SOD1 aggregate conformers developing in the aging CNS, and their ability to propagate templated aggregation to a specific disease-associated mutant variant might influence disease progression and possibly contribute to the heterogeneous phenotypes associated with distinct SOD1-ALS variants. Further inoculation studies and development of the immunohistological analysis of SOD1 conformers in human tissue are ongoing and might shed light on the relevance of distinct SOD1 aggregate strains in ALS patients.

## Supplementary Information

Additional file1.

## Data Availability

All data supporting the findings of this study are available within the submitted Manuscript and the Supplementary Information.

## Abbreviations

ALS: Amyotrophic lateral sclerosis
CNS: Central nervous system
SOD1: Human SOD1
SpC: Spinal cord
SOD1: Superoxide dismutase 1
Tg: Transgenic
aa: Amino acid
BEM: Binary epitope mapping
rbAb: Polyclonal rabbit antibody
mAb: Mouse monoclonal antibody
IHC: Immunohistochemistry
p.i.: Post inoculation
